# Defective Innate Cell Response and Lymph Node Infiltration Specify *Yersinia pestis* Infection

**DOI:** 10.1371/journal.pone.0001688

**Published:** 2008-02-27

**Authors:** Françoise Guinet, Patrick Avé, Louis Jones, Michel Huerre, Elisabeth Carniel

**Affiliations:** 1 Unité des Yersinia, Institut Pasteur, Paris, France; 2 Unité de Recherche et d'Expertise d'Histotechnologie et Pathologie, Institut Pasteur, Paris, France; 3 Groupe Logiciels et Banques de Données, Institut Pasteur, Paris, France; University of Minnesota, United States of America

## Abstract

Since its recent emergence from the enteropathogen *Yersinia pseudotuberculosis*, *Y. pestis*, the plague agent, has acquired an intradermal (id) route of entry and an extreme virulence. To identify pathophysiological events associated with the *Y. pestis* high degree of pathogenicity, we compared disease progression and evolution in mice after id inoculation of the two *Yersinia* species. Mortality studies showed that the id portal was not in itself sufficient to provide *Y. pseudotuberculosis* with the high virulence power of its descendant. Surprisingly, *Y. pseudotuberculosis* multiplied even more efficiently than *Y. pestis* in the dermis, and generated comparable histological lesions. Likewise, *Y. pseudotuberculosis* translocated to the draining lymph node (DLN) and similar numbers of the two bacterial species were found at 24 h post infection (pi) in this organ. However, on day 2 pi, bacterial loads were higher in *Y. pestis*-infected than in *Y. pseudotuberculosis*-infected DLNs. Clustering and multiple correspondence analyses showed that the DLN pathologies induced by the two species were statistically significantly different and identified the most discriminating elementary lesions. *Y. pseudotuberculosis* infection was accompanied by abscess-type polymorphonuclear cell infiltrates containing the infection, while *Y. pestis*-infected DLNs exhibited an altered tissue density and a vascular congestion, and were typified by an invasion of the tissue by free floating bacteria. Therefore, *Y. pestis* exceptional virulence is not due to its recently acquired portal of entry into the host, but is associated with a distinct ability to massively infiltrate the DLN, without inducing in this organ an organized polymorphonuclear cell reaction. These results shed light on pathophysiological processes that draw the line between a virulent and a hypervirulent pathogen.

## Introduction

Bubonic plague is an acute bacterial disease which, if untreated, leads to death in 50–90% of the cases [1] in generally less than 5 days [2]. In spite of major improvements in the management and control of the disease since the ravaging pandemics of the past, the disease is far from eradicated and trends show that it is expanding or re-emerging in many countries [3], [4].

Plague is an anthropozoonosis affecting primarily rodents [2], [5] and the disease has a host-vector-host transmission cycle. The vectors are fleas which transmit the disease by intradermal (id) biting. Humans are generally contaminated through the bite of an infected rodent flea. Experimental id or subcutaneous (sc) injection of the plague agent into laboratory rodents, e.g. guinea pigs, mice and rats, causes a disease similar to naturally acquired bubonic plague [6]–[12]. From the inoculation site the infection proceeds to the draining lymph node (DLN) via lymphatic channels. The infected node increases in size and, within a few days, gives rise to the so-called bubo, hallmark of the disease. Clinically, the bubo is a voluminous and exceedingly painful lymphadenitis, whose sudden appearance coincides with the onset of fever. Histologically, buboes are typically heavily infected and display overwhelming inflammatory and necrotic alterations [6], [8], [13], [14]. Septicemia and hematogenous spread to distal lymph nodes and deep organs appear to follow an initial phase of containment of the infection in the proximal lymph node [11], [15]. The septicemia-induced shock is believed to be the proximal cause of death.

The plague agent is an *Enterobacteriaceae* named *Yersinia pestis*. All other *Yersiniae* are transmitted through the fecal-oral route and they are either non-pathogenic or responsible for generally self-subsiding digestive symptoms [16], [17]. Among the enteropathogenic *Yersinia* species is *Y. pseudotuberculosis*, from which *Y. pestis* originated less than 20 000 years ago [18]. Hence, in a short time frame, *Y. pestis* has developed both an extreme virulence and a mode of transmission unique among the *Enterobacteriaceae* family. The two species are genetically nearly identical [19]. Based on their close relatedness it has been proposed that the two species should be reclassified as a single one, a proposition that was subsequently rejected in consideration of their extreme divergence in pathogenicity, life cycle and public health impact [20], [21]. The *Y. pestis*/*Y. pseudotuberculosis* pair thus provides a unique opportunity to explore, by comparative analysis, the pathophysiological processes associated with a high degree of pathogenicity.

Indeed, the specific mode of action whereby *Y. pestis* rapidly kills its host is still elusive. Little is known of the sequence of events mediating the exceptional severity of plague, including the time and place where these events appear during the course of disease and the impact of the newly acquired dermal portal in plague pathogenesis. To address these questions, we undertook a study of the disease progression of bubonic plague in a mouse model. To highlight pathophysiological events likely to be critical to plague-specific pathogenesis and to identify histological lesions that differentiate plague from other bacterial infections, the findings were contrasted with the disease induced by id inoculation of *Y. pseudotuberculosis*, the closely related and less virulent ancestor of *Y. pestis*.

## Results

### 1. *Y. pseudotuberculosis* is less virulent than *Y. pestis* upon id inoculation

Comparison of the reported lethal doses 50 (LD_50_) of various strains of *Y. pestis* and *Y. pseudotuberculosis* injected sc shows a 10^5^–10^6^ fold higher LD50 for *Y. pseudotuberculosis* [5], [22]–[25]. To test whether a similar difference would appear after id inoculation, which most closely mimics the natural transmission mode of bubonic plague, serial dilutions of *Y. pestis* CO92 and *Y. pseudotuberculosis* IP32953 were injected id into the ear of outbred mice and LD50s were calculated from the mortality rates after a 3 week follow-up. *Y. pseudotuberculosis* LD50 was found to be ∼5×10^5^ colony forming units (cfu), and *Y. pestis* LD50 was ∼20 cfu. Thus, the mortality studies showed that in the id injection model *Y. pestis* CO92 was more virulent than *Y. pseudotuberculosis* IP32953.

Further experiments were carried out to determine the minimum *Y. pestis* infective dose that would cause death of 100% mice and the maximum *Y. pseudotuberculosis* dose to which 100% of the animals would survive. Infective loads between ∼30 cfu to ∼3×10^7^ were tested on a total of 225 mice followed up for a minimum of 7 days. The lowest *Y. pestis* dose to which all mice succumbed within 4 days was between ∼1,000 and ∼2,000 cfu. All mice having received ∼1,300 to ∼17,000 *Y. pseudotuberculosis* cfu were still alive on day 4 post-inoculation (pi) and 2% of them died between day 4 and day 7 pi.

### 2. Bacterial loads at the injection site and in the draining lymph node

To determine whether the different degrees of severity of the *Y. pestis* and *Y. pseudotuberculosis* infections were associated with distinct bacterial dynamics within the host, bacterial loads at the injection site (IS) and in the DLN of *Y. pestis*- and *Y. pseudotuberculosis*-infected mice were quantified. Mice received intradermally (mean±SEM) 4200±427 cfu of *Y. pseudotuberculosis* or 4000±299 cfu of *Y. pestis*. Enumeration of cfu recovered from the IS at time 0 was performed in a subset of experiments to confirm, as previously published [26], that it matched the injected load. Because all of the mice used in the experiments were naive, there were no bacteria in the DLN at time 0. Cfu enumerations from the IS and the DLN were performed 24 h (two groups of 16 mice infected with either *Y. pseudotuberculosis* or *Y. pestis*, 48 h (13 *Y. pseudotuberculosis*- and 18 *Y. pestis*–infected mice), and 72 h and 96 h (2 groups of 7 *Y. pseudotuberculosis*-infected animals each) pi. On day 3 pi, the dramatic decrease in the number of available *Y. pestis*-infected mice, due to around ∼80% mortality, precluded valid statistical analysis of the cfu counts at this time point.

**Figure 1:**
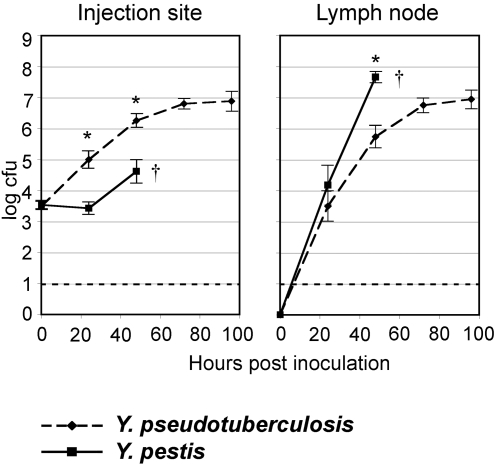
Bacterial loads at injection site and in draining lymph node. Data are means of log_10_ of cfu numbers recovered from 77 mice infected over six independent experiments. Bars = Standard Errors. Stars indicate that the data are significantly different between the two *Yersinia* species (p≤0.0001). The dashed line shows the detection limit (10 cfu). The cross symbol indicates that most *Y. pestis*-infected mice died between days 2 and 3.

*Y. pseudotuberculosis* was able to survive and to proliferate in the dermis during the first two days pi. Its growth rate over the first 24 h pi was even higher than that of *Y. pestis* (Figure 1). Furthermore, *Y. pseudotuberculosis* was found in the proximal lymph node, and similar numbers of both *Yersinia* species were present in the DLN at 24 h (p = 0.39). On the following day, however, *Y. pestis* numbers in the DLN were significantly higher than those of *Y. pseudotuberculosis*. Over extended observation periods of 7 days, DLN *Y. pseudotuberculosis* loads reached a plateau from day 3 on (Fig. 1 and data not shown). Therefore, both species were able to multiply at the inoculation site and to reach the proximal lymph node but while, in the DLN, *Y. pseudotuberculosis* growth was contained, *Y. pestis* multiplication was not controlled and the animals started to die between days 2 and 3.

### 3. *Y. pestis* and *Y. pseudotuberculosis* induce similar lesions at the injection site

In a separate series of experiments, the gross and microscopical pathology at the IS was examined to compare the lesions induced locally by the two *Yersinia* species at 24 h, 48 h and 72 h (for the surviving mice) pi.

**Figure 2:**
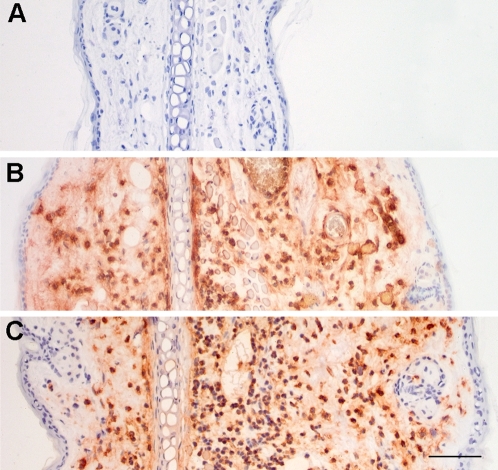
Inflammatory reaction at the injection site. Examples of edema and PMN influx 48 h following injection of ∼4500 *Y. pestis* (panel B) or *Y. pseudotuberculosis* (panel C) cfu, compared to a saline-injected control (panel A). Immunostained PMNs are brown-orange on the preparations. Bar = 20 µm.

At the gross pathology level, the infected ear dorsum appeared normal or exhibited slight inflammatory changes such as a diffuse moderate hyperemia or a red inflammatory dot at the needle entry point irrespective of the injected species. On microscopical examination, edema and polymorphonuclear leukocyte (PMN) influx were observed at the IS of most infected mice. Figure 2 shows examples of PMN reactions at the IS accompanying *Y. pestis* and *Y. pseudotuberculosis* infections. Semi-quantitative scoring of the intensity of the inflammatory response, performed independently by two investigators on a set of 29 infected dermis sections, indicated a slide to slide variation in the intensity of inflammation but no significant impact of the injected species. Thus, *Y. pestis* and *Y. pseudotuberculosis* induced lesions of similar type and intensity at the injection site.

### 4. *Y. pestis* and *Y. pseudotuberculosis* induce different lesions in the draining lymph node

To know whether *Y. pseudotuberculosis* would induce in the proximal lymph node lesions comparable to those observed in the plague bubo, DLNs collected on day 1, 2 and 3 from mice infected with comparable numbers (3,500–6,500 cfu) of either *Y. pestis* or *Y. pseudotuberculosis* were examined. A total of 36 DLNs were analyzed.

#### 4.1. Gross pathology

**Figure 3:**
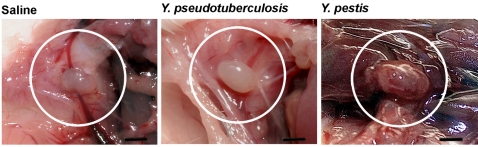
Draining lymph node gross pathology. Macroscopical aspect of lymph nodes two days after inoculation of either saline, ∼5500 *Y. pseudotuberculosis* cfu or ∼5500 *Y. pestis* cfu. Lymph node from *Y. pseudotuberculosis*-infected mice is purulent whereas *Y. pestis*-infected lymph node is reddish and adherent to neighboring tissues. Bar = 2 mm.

While DLNs infected with either *Yersinia* species were macroscopically subnormal on day 1 pi, DLNs collected on days 2 and 3 pi consistently displayed distinctive features of *Y. pestis* or *Y. pseudotuberculosis* infections. *Y. pseudotuberculosis* infected DLNs were frankly purulent, whereas *Y. pestis* infected DLNs were hemorrhagic, sometimes partly purulent, and their consistency was dense and firm (Fig. 3). Therefore, the DLN is the first organ to display species-specific alterations at the gross pathology level during the course of infection, and this difference appears on day 2 pi.

#### 4.2. Histopathology

The lesions present in the infected DLNs were much more complex and varied than in the dermis, so that an objective evaluation of the impact of the species and of the infecting dose on the DLN alterations was not possible without a detailed statistical analysis, using tools suitable for large categorical data sets. Cluster analysis and multiple correspondence analysis were employed, after decomposition of the histological patterns into elementary lesions.

##### a) Cluster analysis identifies histology patterns associated with either Y. pestis or Y. pseudotuberculosis infections

**Figure 4:**
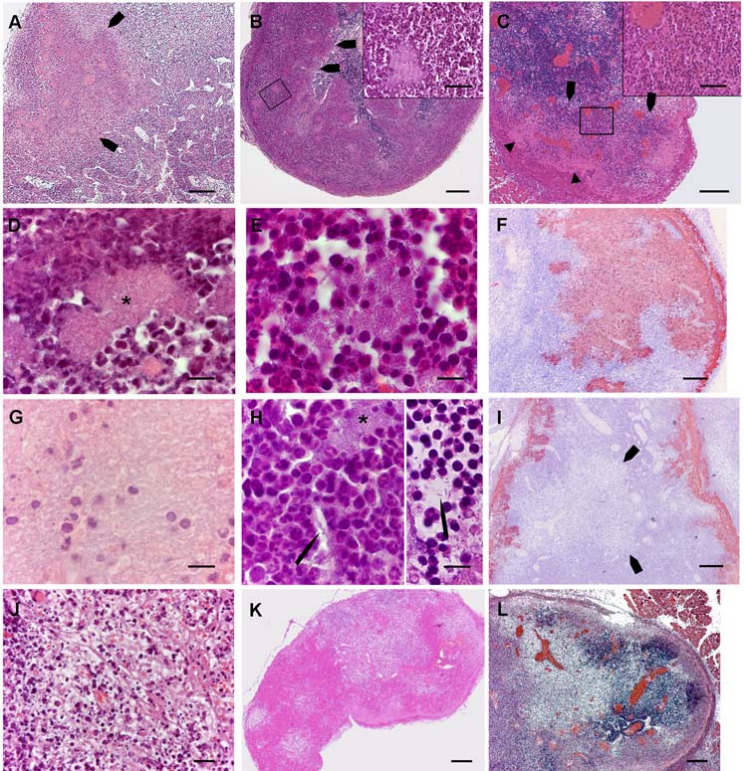
Illustrations of some of the criteria used for scoring. Panels A–E, G, H and J–L display HE stained sections, panels F and I show sections immunostained with *Y. pestis* specific antibodies. In the text below, the criteria are referred to according to the numbering in Table 1. A: Wedge shaped abscess (criterion 4) indicated by arrows. Within the abscess bacterial colonies are visible as pink patches. B: Peripheral layer of PMNs containing bacterial foci (criterion 9), with a blunt demarcation (arrows) from the lymph node tissue (criterion 11). Inset: higher magnification to show the characteristic horseshoe shaped nuclei of the PMNs, and a bacterial focus. C: Layer of PMNs bordering a peripheral band of bacteria, cell debris and PMNs (criterion 10). The inset shows the typical PMN morphology of the cells within the layer. Behind this layer, bacterial aggregates are seen as pink areas (arrowheads) containing purple dots that are, as seen as higher magnification (not shown here) PMNs and cell debris. D: Patch of densely packed bacteria, bordered by PMNs (criterion14). Packed bacteria form a pink 8-shaped area at the centre of the picture (star). E: Atypical bacterial patch (criterion 15). At the center of the picture an aggregate of bacterial rods, not as densely packed as the preceding one, is loosely surrounded by inflammatory cells. F: A large bacterial zone (criterion 17), stained brownish on the preparation. G: Isolated host cells within a bacterial zone (criterion 19). Isolated host cells and cell remnants are seen amid a sea of bacteria which gives a “ground glass” appearance to this part of the LN section. H, *left*: bacterial infiltration around host cells (criterion 20). A bacterial strand (arrow), that seems to originate from a nearby colony (star), passes between host cells. H, *right*: Free floating bacterium, indicated by an arrow (criterion 21). I: Zone of reduced tissular density (arrows) outside bacterial areas, which are brownish on this preparation (criterion 26). This image also shows flame-like inward bacterial projections (criterion 18). J: Area of reduced host cell density with a reticular pattern (criterion 27) and containing numerous pycnotic cells (criterion 32). K: Moth eaten appearance (criterion 33) of a lymph node with areas of contrasting tissular densities. L: Vascular congestion (criterion 40), showing bright red on this preparation. *Magnifications*: Panels A–C, F, I–L : bar = 200 µm. Insets of panels B and C: bar = 50 µm. Panels D, E, G and H: bar = 10 µm.

Criteria aimed at assessing the PMN inflammatory reaction, the extent and organization of bacterial involvement, and changes in DLN morphology and density were defined (Table 1 and Fig. 4), and DLN sections were scored as «+» or «−» for each criterion so that to each DLN was associated a “+/−” sequence representing its histopathological profile and allowing clustering analysis of the data. The scoring was done blinded and repeated at least twice by the same observer for each lymph node.

**Table 1:** Criteria used to score sections of lymph nodes infected with *Y. pestis* or *Y. pseudotuberculosis*, and their discriminating indexes.

General features explored by criteria	Description of criteria	Criterion N°	Illustration on Figure:	Test values
	Similar to saline-injected controls	1		
Extent and organisation of lesions and of inflammatory reaction	PMNs<50% of surface area of the section	2		
	PMNs>50% of surface area of the section	3		
	Wedge shaped abscess	4	4A	−3.19
	Abscess involve one pole of the DLN	5		−3.34
	Abscess>25% of section surface	6		−4.76
	Destructive non-abscess lesions>50% of surface	7		+4.78
	Lesion = 100% of surface	8		+3.95
	Peripheral band of PMNs containing bacterial foci	9	4B	−5.5
	Layer of PMNs bordering a peripheral band of bacteria+PMNs+cell debris	10	4C	+4.75
	Blunt border of inflammatory front	11	4B	−4.46
Extent of bacterial colonization of the LN	Bacteria in sub-capsular sinus, <1/3 of circumference	12		−2.23
	Bacteria: small peripheral focus or foci	13		
	Patches of densely packed bacteria, bordered by PMNs	14	4D	−5.7
	Atypical bact. patches (low density, undefined boundery, weak PMN border)	15	4E	
	Bacteria in ≥ 1/3 of sub-capsular sinus	16		+5.79
	Bacteria involve large areas of the DLN	17	4F	+4.08
Invasiveness in the LN tissue	Inward bacterial projections from the subcapsular sinus (like flames)	18	4I	+5.22
	Isolated host cells within a bacterial zone	19	4G	+6.01
	At high magnification: bacterial infiltration around host cells	20	4H	+6
	At high magnification: free floating bacteria	21	4H	+5.74
Breaching of the PMN barrier	Inward bacterial infiltration beyond the PMN line	22		+5.34
	Bacterial clusters in direct contact with lymphocytes	23		+4.71
Tissue density alterations	Normal LN tissue density	24		−5.22
	Area of reduced host cell density within bacterial areas	25		+4.67
	Area of reduced host cell density outside bacterial areas	26	4I	+4.61
	Area of reduced host cell density with a reticular pattern	27	4J	+3.89
	Area of reduced host cell density without a reticular pattern	28		+4.67
	Area of reduced host cell density in a purulent zone	29		
	Area of reduced host cell density central to the inflammatory front	30		+5.47
	Area of reduced host cell density peripheral to the inflammatory front	31		+2.08
	Presence of numerous pycnotic cells	32	4J	+4.74
	«Moth eaten» appearance	33	4K	+5.97
	Islet of apparently normal lymphocytes in an otherwise altered DLN	34		+4.5
	Part of the DLN is missing	35		
Final stages of LN destruction	Totally destroyed LN massively infiltrated with bacteria	36		
	Totally destroyed LN massively infiltrated with PMNs	37		
Miscellaneaous	Schriveled LN	38		+2.08
	Intranodal haemorrhages	39		+3.27
	Enlarged blood vessels packed with RBCs	40	4L	+5.93
	Surrounding, extranodal tissue, adhering to the LN	41		
	More than 1 LN on the slide	42		

Discriminating indexes are the test values in the right column. Only significant test values (i.e. >1.96 or <−1.96) are given. Positive and negative test-values are distinctive of, respectively, *Y. pestis* and *Y. pseudotuberculosis* infections.

**Figure 5:**
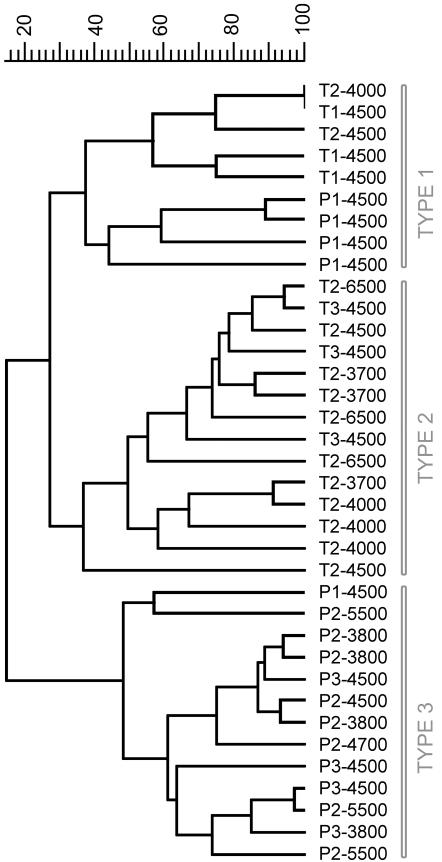
Dendrogram of the draining lymph node histopathological profiles. Each label indicates (in order): infecting species (P for *Y. pestis*, T for *Y. pseudotuberculosis*), delay between mouse infection and lymph node collection (in days), amount of injected cfu. Top scale = % similarity

**Figure 6:**
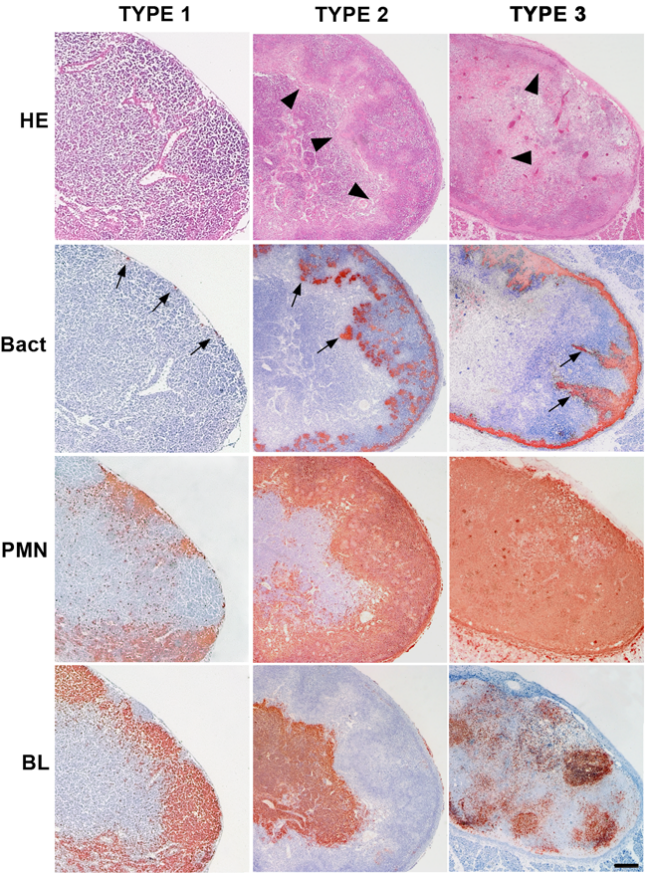
Examples of the three histopathological types of the draining lymph nodes. Lymph nodes were taken 24 h (type 1) and 72 h (type 2 and 3) after inoculation of ∼4500 cfu of *Y. pseudotuberculosis* (types 1 and 2) or *Y. pestis* (type 3). Sections were stained by Hematoxylin-Eosin (HE), and by antibodies specific to *Y. pseudotuberculosis* or to *Y. pestis* (Bact), to PMNs and to B lymphocytes (BL). Immunostainings give a brownish color. Arrowheads on the HE-stained sections indicate the border of the inflammatory front. Bacteria (arrows) are present as tiny bacterial foci at the periphery (type 1), well delimited patches (type 2) or flame-like formations (type 3). The BL staining shows that the overall structure of the type 1, but not of the type 3, lymph node is preserved, while, in the type 2 lymph node, the inflammatory reaction forced B lymphocytes to the central part of the organ. Bar = 100 µm.

The cluster analysis delineated three main types of histopathological patterns (Fig. 5 and Fig. 6). Type 1 patterns corresponded to both *Y. pestis* and *Y. pseudotuberculosis* infected DLNs collected shortly after the bacterial injection. Therefore, this type, which was characterized by no or limited alterations of the lymph nodes, represented early, non species-specific lesions. The type 2 group comprised only *Y. pseudotuberculosis*-infected DLNs, all from day 2 or 3 pi. Hence, type 2 corresponded to advanced *Y. pseudotuberculosis* lesions. Type 3 comprised only profiles associated with *Y. pestis* infection, and all of them but one were from DLNs taken on day 2 or 3 pi. Type 3 thus represented advanced *Y. pestis* lesions. Type 3 DLNs appeared to be generally more severely altered than type 2 DLNs and four of them were totally destroyed, the normal DLN tissue being entirely replaced by bacteria or by PMNs.

Therefore, the cluster analysis strengthened our preliminary observation that a differential evolution of the DLN lesions appears on day 2.

##### b) Exploration of an extended infecting dose range

**Figure 7:**
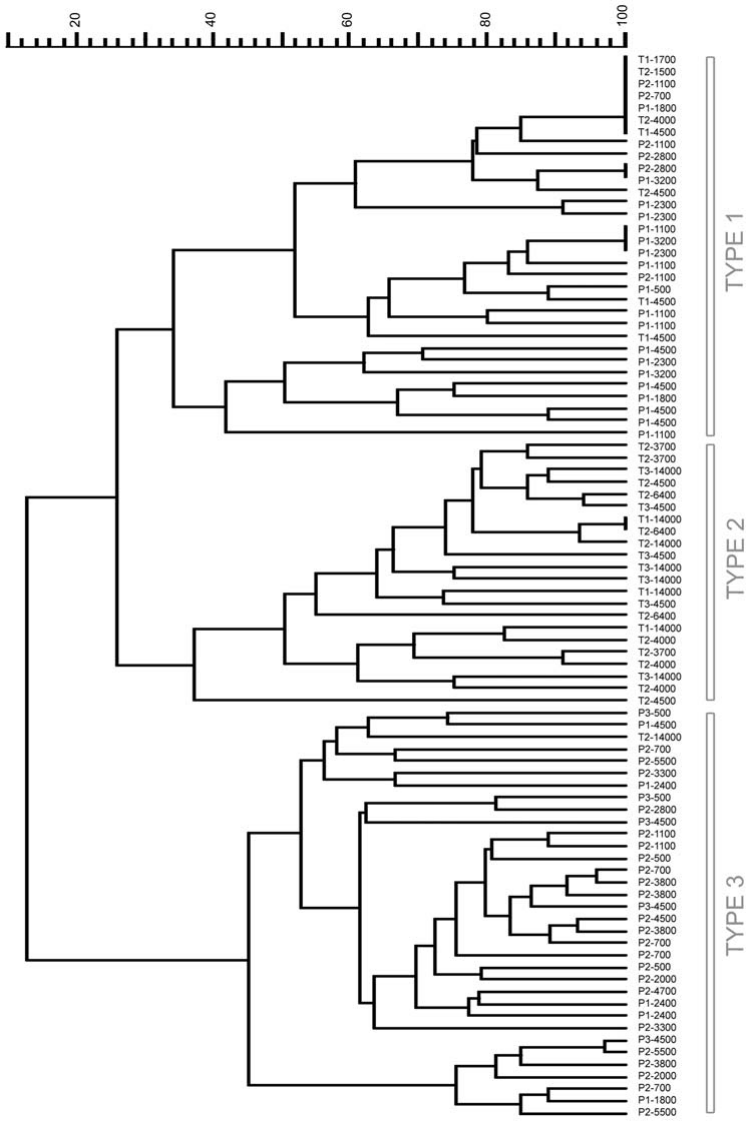
Extended dendrogram of the DLN histopathological profiles. Each label indicates (in order): Infecting species (P for *Y. pestis*, T for *Y. pseudotuberculosis*), time post-injection (in days), amount of injected cfu. Top scale = % similarity. As compared to Figure 5, an extended range of infective-loads was used, and hence more DLNs were included in the analysis (see the Result section). The seven last profiles in the dendrogram correspond to completely destroyed lymph nodes.

An extended bacterial dose range (500–14,000 cfu) was explored, to know whether a higher infecting dose of *Y. pseudotuberculosis* could generate destructive lesions similar to those induced by *Y. pestis*, and if, after infection with lower *Y. pestis* loads, histopathological profiles similar to type 2 would appear. As seen in Fig. 7, this did not modify the relative grouping of infected DLNs, as all DLNs from mice infected with *Y. pestis* segregated in group 1 or 3, while all DLNs but one from mice infected with *Y. pseudotuberculosis* clustered in group 1 or 2. Therefore, the bacterial load does not appear to influence the type of lesions, which is strictly associated with the species type.

##### c) Identification of the criteria most discriminating between types 2 and 3

Thus, the 42 criteria together efficiently discriminated between advanced *Y. pestis* and *Y. pseudotuberculosis* DLN histopathology characteristics. In order to assess the relative contribution of each criterion to this discrimination, lymph node histology patterns were further analyzed by Multiple Correspondence Analysis (MCA), a multivariate statistical method designed to describe objects characterized by categorical variables [27]–[29]. The method reduces the complexity of the initial data set while retaining most of the information on their relationships, and provides graphical outputs so that the above relationships can be visualized. This is achieved through the fitting of axes onto which objects and variables are projected. Here, the «objects» are the DLNs and the «variables» are the criteria. The first axis is calculated as the best fit linear model of the dispersed data, and each successive axis is orthogonal with the preceding axis. For representation of the data, the minimum number of axes are selected that maximizes this representation. All DLNs corresponding to advanced lesions (types 2 and 3) were included in the analysis, except those that were totally destroyed.

**Figure 8:**
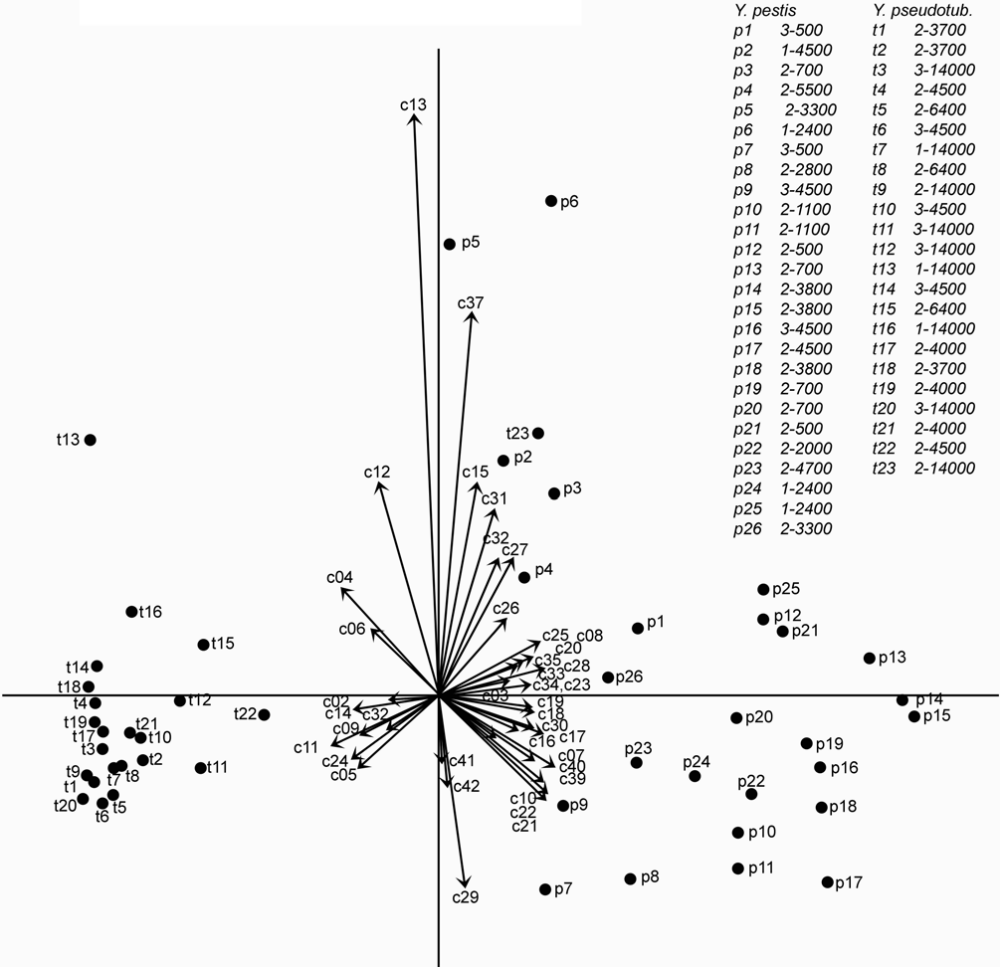
Multiple Correspondence Analysis of type 2 and type 3 histopathological profiles. The types 2 and 3 histopathological profiles represented in the dendrogram of Figure 7, excepted the completely destroyed profiles, were included in the analysis. Here is shown a graphical display of the results of the Multiple Correspondence Analysis, on the first two dimensions. Criteria, numbered as in Table 1, are represented by arrows. Lymph nodes are represented by dots and denoted by alpha-numerical labels which are listed in the accompanying table, at the top right of the figure. In this table are indicated, for each DLN, first, the time interval (in days) between mouse infection and lymph node collection, then, the number of injected cfu. It can be seen that *Y. pseudotuberculosis*- and *Y. pestis*-infected lymph nodes are grouped on the left and right sides of the figure, respectively. Therefore, criteria on the left side of the figure are associated with *Y. pseudotuberculosis* profiles and criteria on the right side of the figure are associated with *Y. pestis* profiles. Criteria that are closest to the first (horizontal) axis are the most discriminating between the two groups.

The first axis, the first two axes together and the first three axes together represented 93.9%, 96.8% and 99.1% of the inertia, respectively, indicating that almost the totality of the information was contained within the first three axes and that the first axis alone contributed most of it. Figure 8 shows a projection on the first two axes of the DLNs with advanced-*Y. pestis* and advanced-*Y. pseudotuberculosis* lesions. It can be seen on the figure that the *Y. pestis* and the *Y. pseudotuberculosis* groups are clearly separated from each other, thus confirming by another method that the two *Yersinia* species induce different alterations of the DLN. Figure 8 also displays the 42 criteria. Criteria located on the left- and right-hand side of the graph are those that characterize *Y. pseudotuberculosis*- and *Y. pestis*-induced lesions, respectively.

To quantify the contribution of each criterion in discriminating between the advanced *Y. pestis* and *Y. pseudotuberculosis* lesions, the “test-value” function of the MCA software was used (see Materials and Methods). The test attributes to each criterion and for each projection axis a numerical value which indicates the probability that the criterion is discriminating between the two groups along this axis. A test-value >1.96 or <−1.96 indicates a >95% probability that the criterion is significantly associated with one of the two groups [30]. Significant test-values for the first axis are shown in Table 1. Positive and negative test-values corresponded to criteria associated with the *Y. pestis* and *Y. pseudotuberculosis* group, respectively.

The criteria most discriminative for *Y. pseudotuberculosis* infection were the patches of packed bacteria in close contact with surrounding PMNs (Table 1, criterion 14) and the peripheral band of PMNs clearly delineated from the DLN parenchyma (criteria 9 and 11). An example of a type 2 DLN exhibiting these features is shown in Fig. 6. Also highly discriminative were images of large size wedge-shaped or polar abscesses (criteria 4, 5 and 6) and a normal tissue density of the organ (criterion 24).

Twenty-three out of 42 criteria were significantly associated with *Y. pestis* infection, reflecting the profound modification induced in the DLN by this bacterium. The highest test-value was that of criterion 19, i.e. images of isolated host cells within bacterial areas. This characteristic might be the result of the ability of bacteria to infiltrate between neighboring host cells, a property corresponding to the second most discriminative criterion for *Y. pestis* infection (criterion 20). A moth eaten appearance, resulting from areas of reduced cell density, was the next discriminating criterion (N°33), followed by vascular congestion (criterion 40). The other criteria having test-values >5 were: large number of bacteria in the sub-capsular sinus, free floating bacteria separated from nearby colonies, the presence of bacteria located centrally to the PMNs, and inward bacterial projections from the subcapsular sinus (criteria 16, 21, 22, 18). Other criteria with lower test values but still strongly characteristic of *Y. pestis* infection corresponded to poorly organized PMN reaction (criterion 10), altered DLN structure and density (criteria 25–28, 32, 34), large extension of the lymph node alterations (criteria 7, 8), breaching of the PMN barrier (criterion 23), high amounts of bacteria (criterion 17) and intranodal hemorrhages (criterion 39). An example of type 3 DLN is shown in Fig. 6. Of note, the two criteria (N°2 and 3) related to the amount of PMNs in the DLN did not discriminate between the advanced lesions induced by the two *Yersinia* species.

## Discussion

Two major steps in the evolution from *Y. pseudotuberculosis* to *Y. pestis* have been the acquisition of an id portal of infection and a sharp increase in virulence, raising the question of a possible causal link between the two events. However, in our system this route of entry did not endow *Y. pseudotuberculosis* with the killing power of its descendant, indicating that the high virulence of *Y. pestis* is a genuine property of this species.

Previous images of dermis sections of human and animal plague cases have indicated that *Y. pestis* penetration into the dermis is followed by local bacterial expansion [8], [31]. This finding is here confirmed and quantified. However, *Y. pestis* dermal expansion is not superior to that of the less virulent species, indicating it does not account for the exceptional severity of plague. In fact, unexpectedly, there were even higher numbers of *Y. pseudotuberculosis* than *Y. pestis* within the IS at 24 h and 48 h pi. This difference does not seem to be linked to a lower in situ inflammatory reaction to *Y. pseudotuberculosis* because similar inflammatory lesions were observed in the dermis of mice injected with either *Yersinia* species. At 24 h pi, similar numbers of *Y. pestis* and *Y. pseudotuberculosis* were present in the DLN. Altogether these observations show that the first steps of the disease, including a phase of bacterial multiplication in the DLN within the first 24 h, proceeded similarly during the two *Yersinia* infections, indicating that the expression of *Y. pestis* unique pathogenicity is delayed until later stages of the infectious process.

In contrast, *Y. pestis* loads in the DLN were higher than those of *Y. pseudotuberculosis* on day 2, indicating that, in this organ, *Y. pestis* growth was not controlled as efficiently as that of *Y. pseudotuberculosis* past the first 24 h of infection. Our findings show that the large accumulation of bacteria in the draining lymph node previously reported in descriptions of human and animal plague buboes [6], [8], [13], [14] is specific to *Y. pestis* compared to its sister species. As has been suggested, accumulation of *Y. pestis* in the extravascular lymph node reservoir, followed by brutal release into the blood stream, may be important for the generation of the high level septicemia necessary for transmission to a new host by the blood sucking vector [22]. Therefore, the difference in the intranodal bacterial accumulation of the two species might be critical to their different pathogenic potentials.

Significantly different histopathological patterns were associated in the DLN with *Y. pestis* and *Y. pseudotuberculosis* infections. This conclusion was reached through decomposition of images of the histological sections into elementary lesions and subsequent analysis by statistical tools able to deal with large sets of data with no assumed theoretical distribution of the variables or the individuals [27]–[29]. To our knowledge the approach used here, which combines the use of clustering analysis methods, MCA and test-value calculations, has never been reported so far for comparative analysis of histological images. While this study focused on the host response to *Yersinia* infection, another potential application of this approach is the comparison of the lesions induced by *Y. pestis* mutants in order to determine the role of specific genes to plague pathogenesis. These mutants would likely express phenotypes less distant from *Y. pestis* than does *Y. pseudotuberculosis*. However, the technique was developped to detect small differences in complex data sets so it could probably detect differences more subtle than between the two *Yersinia* species, although it would probably give lower percentages of inertia for each axis.

The MCA analysis yielded a list of elementary lesions that specified the two types of infection with respect to one another. All the abscess-type lesions, whether wedge-shaped, polar or organized as a peripheral band, were characteristic of *Y. pseudotuberculosis* infection and, in these structures, the infectious foci were kept separate from the normal lymph node tissue. Thus, an organized PMN response able to contain the bacteria was a hallmark of *Y. pseudotuberculosis* infection, which implies, conversely, that one specificity of plague lesions was the lack of an organized innate cell reaction. Indeed, PMNs in buboes were often disposed as a thin, irregular and fragmented layer, a feature highly discriminatory towards plague. Abscesses are the expression of an acute cellular response to bacterial invasions and play a major role in the control of bacterial infections [32]. Therefore the lack of an abscess-type structure is certainly a major impediment to the control of *Y. pestis* proliferation in the DLN and probably accounts largely for the high representation of plague bacilli in this organ.

The molecular and cellular mechanisms underlying abscess formation remain poorly defined [33], [34], except for the step of PMN recruitment from the blood stream about which several inflammatory cues and molecular mechanisms have been uncovered [35], [36]. The *Y. pestis*-associated deficit in abscess formation is not the result of a massive defect of PMN recruitment to the DLN, because, from the morphological data, there was no gross difference in the amount of PMNs in the DLN during the two *Yersinia* infections. Therefore it is possible that functional alterations of the PMNs, or of other cells involved in abscess formation, are induced by the plague agent.

Bacterial extensions from the subcapsular sinus or from colonies, seen at low magnifications as «flames» and at high magnification as bacterial strands streaking between host cells, and free floating isolated bacteria, all reflected an infiltrating character of the plague infection. The images of isolated host cells within bacterial areas might be the result of this infiltrating process around host cells. The above criteria all had discriminatory values above 5, making the infiltrative character of the infection the most specific feature of plague in the comparison with *Y. pseudotuberculosis* infection. It remains to be determined whether specific mechanisms, such as selective tissue destructions, are responsible for this infiltrating behavior, in addition to the failure of the inflammatory cells to contain the infection.

In conclusion, no major differences were noted between the two *Yersinia* infections during the progression to DLN, and in the DLN before day 2 pi. At this point, *Y. pseudotuberculosis* infection had induced an organized PMN reaction and was contained, unlike *Y. pestis* growth which was not controlled, leading to death within hours. These findings point to the population of PMNs recruited to the DLN as a likely primary or secondary target of the *Y. pestis* specific strategy. More work directed at characterizing the mechanisms by which *Y. pestis* specifically prevents the formation of an effective abscess-type defense should further our understanding of plague pathogenesis.

## Materials and Methods

### Animals and bacteria

Eight-week old female OF1 mice (Charles River, l'Arbresle, France) were maintained under specific pathogen-free conditions at the Institut Pasteur in compliance with European animal welfare regulations. Bacterial strains were *Y. pestis* CO92 [37] and *Y. pseudotuberculosis* IP32953 [19]. Cultures were carried out at 28°C on LB agar medium supplemented with 0.002% (w/v) hemin. Prior to animal infections, bacteria were resuspended in saline and concentrations of the resulting suspensions were verified by plating on agar medium. Mice were anaesthetized by intraperitoneal injection of 0.5 ml Avertin (1.25% Tribromoethanol+2.5% tert-amyl alcohol) and the ear dorsum was spread on several layers of adhesive tape applied on the experimentator thumb, so as to facilitate the injection and protect the experimentator from accidental self-injection. In preparatory studies, it was verified by id injection of 1% methylene blue that the draining lymph node of the ear dorsum was the superficial parotid lymph node, according to the nomenclature defined in [38]. For infections, ten microliters of bacterial suspensions were injected id with a 0.3 ml insulin syringe (Becton Dickinson, New Jersey, USA). At various time points, infected ears and ipsilateral draining lymph nodes were collected and crushed, and recovered bacteria were enumerated by plating on agar medium. LD_50_ were estimated according to the method of Reed and Muench [39], with groups of 5 mice followed up for 21 days. Protocols for animal experiments were prepared according to the guidelines of the Institut Pasteur safety committee.

### Histology and immunohistochemistry

Skin samples at the inoculation site and draining lymph nodes were fixed in 4% neutral buffered paraformaldehyde, and processed by usual methods with standard hematoxylin-eosin (HE) staining [40]. To identify inflammatory and immune cells, samples were collected in a Zinc-based preservative [41], [42] and then in alcohol before being embedded in low melting-point paraffin (Poly(ethylenglycol) diesterate, Aldrich). Sections were treated for endogenous peroxidase activity by incubation for 20 min in 0.3% (v/v) H_2_O_2_ and blocked for 20 min in normal serum from the appropriate animal host (dilution 1∶10 in PBS (pH 7.4) containing 1% (w/v) milk powder) prior to incubation for 1 h with one of the following antibodies: a rabbit anti huCD3, which cross reacts with mouse CD3 antigen (pre-diluted; NeoMarkers), a rat anti-mouse CD45R (B220 clone, dilution 1∶40; Caltag), a rat anti-mouse F4/80, (dilution 1∶50; Caltag), a rat anti-mouse GR1 (dilution 1∶200, Caltag), a hamster biotinylated anti-mouse CD11c (dilution 1∶40; BD Pharmingen), and rabbit polyclonal antisera specific for the *Y. pestis* F1 Ag, and the *Y. pseudotuberculosis* type I O-Ag (produced by the French Reference Center for *Yersinia*). After three washes in PBS-1% (w/v) milk powder, sections were incubated for 1 h with appropriate secondary antibodies: EnVision+System-HRP Anti-Rabbit (undiluted, Dako), streptavidin-peroxidase conjugate (diluted 1∶600, Dako), or rat-specific biotinylated Ig (diluted 1∶400) followed by streptavidin-peroxidase conjugate. Bound peroxidase activity was detected using 3-amino-9- ethylcarbazole (AEC) substrate (Sigma) [41], [43]. Tissues were counterstained with Harris' hematoxylin.

Slides were examined through an E800 Nikon microscope equipped with 2× to 100×Plan Apochromat objectives. Determination of the criteria for scoring was made upon examination of preparations stained by HE and with all the above antibodies; scoring was made on preparations stained by HE and with anti-*Y. pestis* or anti-*Y. pseudotuberculosis* antibodies. Sections were identified by a code that did not reveal which *Yersinia* species had been injected.

### Statistical analysis

#### *S*ignificance tests for bacteria enumeration data

Series of injected *Y. pestis* and *Y. pseudotuberculosis* cfu numbers and bacterial loads in organs were compared using the InStat software (GraphPad Software, San Diego California USA). Log-transformed data were analyzed. The software uses Kolmogorov-Smirnov and Fisher tests to verify the normality of the data distribution and the homogeneity of variances. Depending on the results of these tests, unpaired t-tests or Man-Whitney tests were performed.

#### Clustering analysis

Cluster analysis of the histology patterns was performed with the BioNumerics software, version 4.0 (Applied Maths, Kortrijk, Belgium) using the unweighted pair group method with average linkages (UPGMA) and the Dice coefficient to analyze the similarities of the patterns.

#### Multiple Correspondence Analysis (MCA)

The MCA was performed on a complete disjunctive form table, using the software SPAD [30] and the ade4 [44] package for the R statistics system. The SPAD software was also used to calculate test-values [30], which are numerical indicators of the influence of a variable for discriminating defined subgroups of objects. Calculation of the relative contribution of each axis to the total inertia was done according to the formula adapted by Benzécri for use on complete disjunctive form tables [45].
